# Group A Streptococcal Invasive Infections Among Children in Cyprus

**DOI:** 10.3390/microorganisms13081783

**Published:** 2025-07-31

**Authors:** Maria Koliou, Gavriella Ioannou Vassiliadou, Athina Aristidou, Petros Ladas, Andreas Sergis, Maria Argyrou, Myria Charalambous, Markella Marcou, Maria Alexandrou, Juliana Coelho, Yan Ryan, Androulla Efstratiou, Stella Mazeri

**Affiliations:** 1 Medical School, University of Cyprus, Nicosia 2115, Cyprus; gavriella.v@outlook.com (G.I.V.); ladas.petros@ucy.ac.cy (P.L.); 2 Archbishop Makarios Hospital, Nicosia 2012, Cyprus; athina.aristeidou@hotmail.com (A.A.); sergiandreas@hotmail.com (A.S.); argyrou.maria@yahoo.com (M.A.); myriach55@gmail.com (M.C.); markella.marcou@hotmail.com (M.M.); 3 Larnaca General Hospital, Larnaca 6043, Cyprus; mralexandrou@gmail.com; 4 UK Health Security Agency, London NW9 5EQ, UK; juliana.coelho@ukhsa.gov.uk (J.C.); yan.ryan@ukhsa.gov.uk (Y.R.); androulla.efstratiou@ukhsa.gov.uk (A.E.); 5 Roslin Institute, Royal (Dick) School of Veterinary Studies, University of Edinburgh, Edinburgh EH25 9RG, UK; stellamazeri@gmail.com

**Keywords:** invasive group A streptococcal infections, children, scarlet fever, streptococcal toxic shock syndrome

## Abstract

An increase in invasive group A streptococcal (iGAS) infections among children under 15 years of age was reported in several countries between late 2022 and early 2023. This retrospective study aims to describe the epidemiology and clinical features of iGAS infections in children in Cyprus during the same period. Medical records of patients under 16 years old admitted with iGAS infection to the Archbishop Makarios Hospital, the only tertiary paediatric referral centre in Cyprus, between 1 January 2021 and 30 June 2024, were reviewed. Twenty-two cases were identified, of which twenty were classified as confirmed and two as probable. Half of the cases occurred in children aged 0–4 years, and 59% were recorded between December 2022 and April 2023. Scarlet fever was diagnosed in six children, five of whom developed pneumonia and empyema. Streptococcal toxic shock syndrome (STSS) was observed in five patients, resulting in two deaths and one case requiring prolonged extracorporeal membrane oxygenation (ECMO). The overall case fatality rate was 9.1%. *Emm* 1, belonging to the M1UK clone, was the predominant strain (66.6%). The findings underscore the severity of iGAS infections, particularly in younger children, and highlight the importance of timely diagnosis, appropriate management and continued epidemiological surveillance.

## 1. Introduction

Beta haemolytic group A *Streptococcus* (GAS), also known as *Streptococcus pyogenes* or Lancefield group A *Streptococcus*, is an aerobic Gram-positive coccus that can cause a wide range of infections. These include superficial infections such as pharyngotonsillitis, skin and soft tissue infections, and scarlet fever, as well as severe invasive diseases with substantial morbidity and mortality. Post-infectious complications, such as rheumatic fever and post-streptococcal glomerulonephritis, can also occur [1]. Invasive group A *Streptococcus* (iGAS) infections are defined by the invasion of normally sterile sites and can present with various clinical syndromes, such as bacteraemic pneumonia, with or without empyema, primary bacteraemia and deep soft tissue infections, including necrotising fasciitis. Streptococcal toxic shock syndrome (STSS) occurs in approximately one-third of cases and is associated with high mortality due to toxin release, leading to multiorgan failure [2].

Confirmed cases of iGAS disease are defined when GAS is isolated from a sterile site, such as blood, cerebrospinal fluid or pleural fluid [2]. Probable cases are defined as clinically severe illness (e.g., septic shock, STSS or necrotising fasciitis) where GAS is isolated from a nonsterile body site (e.g., throat, sputum, wound) and no other bacterial pathogen is isolated [2].

Risk factors for invasive GAS infections include skin lesions, chronic illnesses, prior or concurrent viral infections such as influenza or varicella and age [3,4]. Specifically, iGAS infections have a high incidence rate among children under the age of 4 and adults over the age of 65 [3,5,6,7,8]. In temperate regions of the northern hemisphere, such as in Europe and the US, iGAS infections follow a seasonal pattern, peaking between December and April, although cases may occur throughout the year [3,6,7]. Even so, an increase in iGAS cases and related deaths was reported across several countries in late 2022 and early 2023, with a particularly notable rise observed in children under 15, especially in the 0–4-year age group [5,6,7,8,9,10]. These countries include England, the Netherlands, Denmark, France, Spain, Sweden and the US [5,6,7,8,9,10].

In the Republic of Cyprus, iGAS infection only became a notifiable disease in December 2022. As a national referral centre with paediatric intensive care services, Archbishop Makarios III hospital receives severe cases of iGAS from all over the country. The aim of this study is to describe the epidemiology and characteristics of invasive GAS infections in children admitted to our hospital between January 2021 and June 2024.

## 2. Materials and Methods

### 2.1. Setting

This study was performed at the Archbishop Makarios III hospital, which is the national paediatric tertiary care referral centre of the Republic of Cyprus, located in Nicosia, the capital of Cyprus. The hospital serves a catchment population of 56,444 children under 16 years in the Nicosia district. The population source is the 2021 Population Census conducted by the Statistical Service, as well as the annual demographic reports [11,12]. The hospital is also the national referral centre for severe paediatric infections, including iGAS cases, as it is the largest facility with all paediatric subspecialties and the only one with paediatric intensive care services. The study period was set from January 2021 to June 2024, based on the initiation of more systematic case recording at our hospital.

### 2.2. Study Design

We retrospectively reviewed the data from medical records of paediatric patients <16 years admitted with iGAS infection at the Archbishop Makarios III hospital in Nicosia, Cyprus, between 1 January 2021 and 30 June 2024. Records were retrieved from the electronic admission coding system using a predefined comprehensive list of terms including ‘invasive GAS disease’, ‘complicated pneumonia by group A *Streptococcus*’, ‘bacteraemia by GAS’ and ‘otitis media complications by GAS’ as the final discharge diagnosis. The search was performed using a text-based search to identify potential cases. No additional laboratory audit was performed beyond the admission coding search. Patients with probable or confirmed invasive GAS disease were included in the study. A confirmed case of invasive infection was defined as disease with isolation of GAS by culture or molecular method (PCR) from a normally sterile body site, such as blood, CSF or pleural fluid, as identified by the Microbiology Laboratory of Archbishop Makarios III Hospital using the standard culture and PCR protocols described below. A case of probable invasive GAS infection is defined as a clinically severe illness, such as maternal sepsis, septic shock, STSS or necrotising fasciitis, for which no other bacterial aetiology has been identified and in which GAS is isolated or detected from a nonsterile site (e.g., throat, sputum, wound, superficial skin abscess, subcutaneous tissue or placenta) [2]. The clinical criteria for STSS included hypotension and multiorgan failure [13].

### 2.3. Collection of Patient Data

Data regarding age, gender, admission date, duration of hospitalization and district of origin were retrieved. Information on the clinical presentation, management and outcome of the case was also collected. In addition, any predisposing factors reported were collected, e.g., diabetes, cancer, HIV, chronic lung or heart disease, immunocompromising condition, varicella or influenza—recent or concurrent infection. Finally, information on the GAS strains was collected, such as (A) site of GAS detection (blood, joint fluid, cerebrospinal fluid (CSF), pleural fluid, peritoneal fluid, abscess,); (B) antibiogram; (C) *emm* type; and (D) genomic relationships between the strains.

### 2.4. Isolation of GAS

For blood culture, 1–3 mL of blood from the patient was inoculated into BD Bactec Peds Plus/F Culture Vials (Plastic) at the ward. In the Microbiology Laboratory, the vials are incubated in the BD Bactec FX Blood Culture System (Becton, Dickinson and Company, Macquarie Park, NSW, Australia) for up to five days. When a positive culture is detected, the vial is removed from the BD Bactec FX Blood Culture System. A Gram stain is performed as well as a direct antibiogram according to the result of the Gram stain. Based on the Gram stain and clinical context, positive blood cultures were subcultured onto appropriate solid media, including Blood Agar, Chocolate Agar, McConkey Agar, Sabouraud Agar and Schaedler Agar. Blood Agar, McConkey Agar and Sabouraud Agar are incubated aerobically, Chocolate Agar is incubated in 5–10% CO_2_ and Schaedler Agar is incubated anaerobically at 37 °C for 24 h and then examined for growth. GAS is identified by typical colony appearance on Blood Agar and the use of the Prolex Streptococcal Grouping Latex Kit (Pro-Lab Diagnostics, Fisher Scientific, Loughborough, UK). Sensitivity testing of GAS is performed by the disc diffusion method according to the EUCAST guidelines [14].

Pleural and synovial fluids are sent to the Microbiology Laboratory in sterile containers without additives. Culture is performed using the deposit. The specimen was centrifuged, and the resulting deposit (sediment) was used to inoculate solid media plates (Blood Agar, Chocolate Agar, McConkey Agar, Sabouraud Agar, and Schaedler Agar) as well as Brain Heart Infusion broth. Blood Agar, McConkey Agar, Sabouraud Agar and Brain Heart Infusion are incubated aerobically, Chocolate Agar is incubated in 5–10% CO_2_ and Schaedler Agar is incubated anaerobically at 37 °C. The rest of the process is as described above for the blood cultures.

### 2.5. Molecular Identification of GAS by Real-Time PCR

The real-time PCR protocol for the detection of GAS in patient samples (whole blood/pleural fluids) involves DNA extraction followed by real-time PCR amplification. DNA extraction of both whole blood and pleural fluid samples was achieved using the QIAGEN UCP Pathogen kit (QIAGEN, Aarhus, Denmark). Detection of GAS was achieved by real-time PCR, using a Rotor Gene Q real-time PCR machine (QIAGEN, Aarhus, Denmark). Primers and probes used in this protocol are described by Kodani et al. [15].

### 2.6. Molecular Analysis of Isolated GAS Strains

Nine of the isolated strains were saved and submitted to the *Streptococcus* Reference laboratory, UK Health Security Agency (UKHSA, London, UK), for *emm* typing and whole genome sequencing. WGS sequencing of Streptococcus pyogenes isolates and relatedness analysis was performed as described in Vieira et al. [16].

### 2.7. Statistics

Data were analysed using R statistical software version 4.2.0 [17]. Package ggplot2 was used for plotting [18].

## 3. Results

A total of 22 cases of invasive GAS disease were identified between 1 January 2021 and 30 June 2024 in children under the age of 16 who were admitted to Archbishop Makarios III Hospital. Two of these cases were defined as probable and twenty were confirmed iGAS cases. Patient demographics were as follows: Among patients, 50% (11/22) were female. The median age of patients was 4 years (interquartile range 2.0–6.6), with 50% (11/22) being in the age group of 0–4 years old. Sixteen patients were Cypriots, while the remainder were of foreign nationality. Most were known to be residing in Cyprus at the time of admission. Symptoms on admission included fever in all cases, scarlatiniform rash in 27.3% (6/22), cough in 36.4% (8/22), otalgia in 27.3% (6/22) and diarrhoea in 18.2% (4/22).

**Figure 1:**
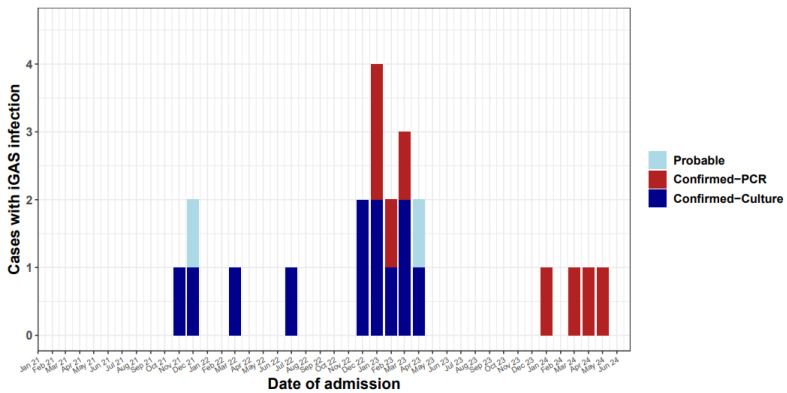
Invasive GAS cases in children < 16 years old in Cyprus by month, 2021–2024.

A total of 59.1% (13/22) of cases occurred between November 2022 and April 2023, while 18.2% (4/22) of cases occurred between November 2021 and April 2022. Four cases were also detected between November 2023 and June 2024, with eight cases diagnosed by PCR and fourteen confirmed by culture of blood or other biological fluids (Figure 1). Table 1 describes the isolation sites for all confirmed and probable cases. Most were blood (31.8%) and pleural fluid (31.8%). In one case GAS was isolated from both blood and joint fluid. In the two probable cases GAS was isolated from perforated ear pus and from throat swabs.

Clinical syndromes caused by iGAS strains and outcomes are described in Table 2. In eight cases, the infection led to pneumonia with empyema (36.4%). Seven cases presented with otogenic complications (31.8%), which included bacteraemia/perforated ear drum to mastoiditis, meningitis and sigmoid sinus thrombosis. Five of the six cases of scarlet fever were further complicated with pneumonia and empyema. Two of the fifteen cases included deep soft tissue infections (fasciitis). Overall, seven patients required hospitalization in the intensive care unit. STSS was present in five cases. Two of these cases died and one required prolonged extracorporeal membrane oxygenation (ECMO) support. The two patients who died were 16 and 26 months old. Both patients died within hours of admission from multiorgan failure. The overall case fatality rate in our series was 9.1%.

Predisposing factors for iGAS infections were reported in five cases (22.7%) as follows. Two patients tested positive for influenza and one for metapneumovirus. These three patients developed pneumonia with empyema. One case with ventricular septal defect was complicated with bacteraemia/endocarditis. Another case, with superficial wound infection as a predisposing factor, also developed cellulitis/fasciitis. All children were negative for SARS-CoV-2, following routine testing during their admission to the hospital.

### 3.1. Antimicrobial Resistance

Sensitivity testing by disc diffusion was performed on 13 iGAS strains. They were universally (100%) susceptible to penicillin. One strain (7.7%) was resistant to erythromycin, and showed inducible resistance to clindamycin, as documented by a positive D-zone test. Susceptibility to tetracycline was available for 10 strains. Two of them were found resistant (20%).

### 3.2. Emm Types

Typing data were available for nine of the fifteen isolated GAS strains and are presented in Table 3. *emm* 1, including subtypes *emm* 1.0 and *emm* 1.150, was the most predominant type detected in seven strains tested (77.8%); *emm* 22 was implicated in one of the deaths. In the case which required ECMO the GAS strain isolated was *emm* 1 type. Whole genome sequencing data revealed that all the *emm* 1 GAS strains belong to the M1UK clone reported from the UK [19].

**Figure 2:**
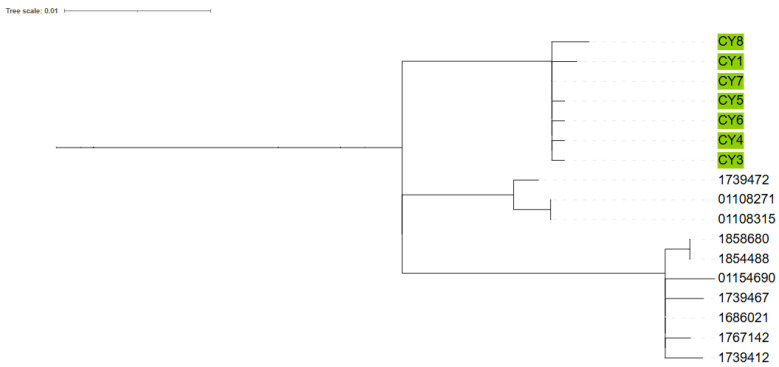
Phylogenetic tree comparing Cyprus samples to contemporary UK M1_UK_ isolates.

Whole genome sequencing of the isolates additionally showed a median of two single-nucleotide polymorphism (SNP) differences among Cypriot isolates (interquartile range [IQR]: 2). When compared to contemporary UK isolates, the median number of SNP differences was 25 (IQR: 32). Intriguingly the *emm* 1.150 isolate is only 3–5 SNP differences from standard *emm* 1.0 and therefore is potentially a mutation of *emm* 1.0 from the same ancestor rather than an exogenous *emm* 1.150, which only requires a single SNP difference in the *emm* gene. All Cypriot isolates, when compared to a selection of UK-based background *M1UK* isolates, were found to have median SNP distances of 24.4 and 36, respectively, to the two nearest UK clades (Figure 2). The UK isolates shown in the figure represent contemporary clinical isolates from 2022 and 2023, and are included for phylogenetic context. Clusters among Cypriot isolates are indicated by colour coding. Five of the seven *emm* 1 strains appeared to be very closely genetically related and were all from cases that did not have an obvious epidemiologic link between them, other than being residents in and around the Nicosia district. The other two *emm* 1 strains, which were slightly more genomically distant, but still closely related to the other strains, were from cases who resided in other districts of Cyprus.

## 4. Discussion

Our study revealed an increase in iGAS infections among children in Cyprus during the period November 2022 to April 2023, in parallel with the trends observed in other European countries and the US [3,5,6,9]. We have reviewed all iGAS cases under 16 years old admitted to the referral tertiary paediatric centre the Archbishop Makarios Hospital from January 2021 to June 2024. A total of 13 out of 22 iGAS cases occurred between November 2022 and June 2023, compared to just 4 cases in each of the previous and subsequent winters.

The median age of affected children was 4 years, with 50% belonging to the 0–4-year age group, aligning with reports from other countries [10]. Interestingly the percentage for female patients was equal to that of males in our series of patients (50%), in contrast to the reports from other countries, including England, Denmark and the US, where there was a slight male predominance [6,9,19].

Although the first case was detected in November 2021, the notable increase in invasive GAS cases in Cyprus began in mid-December 2022 and continued until the end of April 2023, in contrast to other countries where increases were reported earlier in 2022 [19,20]. This may be due to climatic differences between Cyprus and the northern countries, namely warmer weather, which is also reflected in a delayed seasonal influenza pattern in Cyprus, which usually starts at the end of December of each year and extends towards the end of April. The role of co-infecting viruses in the severity of these infections is well recognized in many countries, such as in England, the Netherlands and the US [7,8,19].

The most frequent clinical form was pneumonia with empyema, and more than 30% of cases had otogenic complications. Scarlet fever was present in six cases, most of which were complicated by pneumonia, mirroring trends seen in the UK [21,22]. STSS is a well-documented complication of iGAS disease carrying a high mortality rate of 30–79% [3,15,23,24]. STSS was present in five cases in our series, and two of them had a fatal outcome within a few hours of admission to the intensive care unit. The third case had a serious course of disease with concurrent pneumonia and empyema and required prolonged ECMO support. Seven of the patients required intensive care for at least a few hours (ranging from 5 h to one month). The fatality rate of 9.1% in our series of cases appears high in comparison to some other countries. Predisposing factors for invasive disease were recorded in five cases. Three were initially diagnosed with influenza or metapneumovirus infection, and these cases were further complicated with pneumonia and empyema. Unfortunately, information on concurrent viral infections was incomplete in many cases.

Antimicrobial susceptibility testing for antibiotics on 13 GAS strains revealed that the majority of strains were sensitive to the four antibacterials tested. Isolates were universally sensitive to penicillin. One strain was found resistant to erythromycin (7.7%) and displayed inducible resistance to clindamycin. Tetracycline resistance was identified in two out of ten strains (20%), which is similar to the strains from England (25%) [25], and also to the rate (18%) detected in GAS strains from pharyngeal infections in children in Cyprus in 2003–2004 [26]. Resistance to erythromycin appears to have increased compared to the 2003–2004 study where it was found to be very rare at just 1.1%. This might be due to the introduction and extensive use in Cyprus of the azalide azithromycin for the treatment of respiratory tract infections in children since 2005 [26,27].

*Emm* 1 was the most predominant type (77.8%) of the strains typed, and this is similar to the findings from England and Denmark (55% and 57%, respectively) [6,25]. In Australia, the most common *emm* type was also *emm* 1, but with a lower rate, 37% [28]. In the Netherlands, a specific and dominant *emm* type was not observed amongst invasive infections. Instead, several types such as 1, 4, 12, 22 and 89 accounted for more than 80% of invasive cases in this population [5]. In the series of cases described in Texas, US, *emm* type 12 appeared to be the most frequent type, in contrast to the prepandemic period when *emm* 1 was the dominant type [9].

Whole genome sequencing revealed that the M1 strains tested from Cyprus belonged to the M1UK clone. This agrees with the findings from the UK [20] and Iceland [6]. The M1UK clone was reported in the UK during the last decade and has been associated with scarlet fever cases as well as invasive cases of GAS in the UK [21]. The dissemination of the M1UK clone was reported in many countries, such as Canada, Australia and Denmark [21,22,29]. This is the first time this clone has been detected from Cyprus. In Denmark, M1UK was the predominant clone in the prepandemic period. However, in the recent surge of iGAS infections a new clone has emerged, the M1DK clone, which accounted for approximately 30% of recently sequenced M1 strains in Denmark [6].

One hypothesis for the possible aetiology of this resurgence of invasive GAS infections is the adoption of non-pharmacological interventions (NPIs) such as the face masks and the social distancing (e.g., lockdowns) imposed during the COVID-19 pandemic in order to control the spread of the SARS-CoV-2 virus. This led to a decrease in the burden of many paediatric infections caused by viruses and bacteria, especially during the first year of the COVID-19 pandemic [30]. This lack of exposure to pathogens might have prevented immune system stimulation and development of immunity against many pathogens. This increased the proportion of susceptible people among the population and decreased herd immunity to many pathogens, including group A *Streptococcus*. The substantial increase in the burden of viral pathogens such as RSV and especially influenza virus after the lifting of NPIs may be another factor which contributes to the increase in many invasive bacterial diseases such as invasive pneumococcal disease and invasive GAS disease [5,8,31,32]. In England the unusual increase in scarlet fever activity during 2022 was also considered one of the factors contributing to increased invasive GAS disease [25].

A limitation of this study is its retrospective nature, which may have led to missing data on the cases or on predisposing factors. However, there is much clinical information reported in the notes, which allows for clinical analysis of the cases. A second limitation is that iGAS infections were not a notifiable disease in the Republic of Cyprus until December 2022. Therefore, it is not easy to draw conclusions on an increase in cases in comparison to the prepandemic years. However, our hospital’s electronic information/archive system allows comprehensive case identification and analysis. Another limitation of the study is that patients were not routinely screened for other viruses except for SARS-CoV-2. Therefore, the role of other viruses such as RSV and influenza as predisposing factors cannot be fully evaluated. Additionally, molecular analysis was not possible for all isolated strains; however, among those strains analysed, *emm* 1 type was clearly predominant.

## 5. Conclusions

Increased numbers of iGAS disease cases were detected in Cyprus from December 2022 to April 2023. The high incidence of iGAS in children under 4 years of age as well as the mortality associated with STSS emphasize the severity of iGAS infections and the need for timely diagnosis, treatment, prophylaxis and epidemiological surveillance. Epidemiological investigations including screening of contacts and notification of cases may contribute to effective control of these severe infections which carry substantial morbidity and mortality. Public education on prophylactic measures, including influenza and varicella vaccination, is of great importance. Further characterization of strains isolated from these invasive infections, such as *emm* typing and whole genome sequencing, will help detect outbreaks and the origin of strains, and also monitor trends as well as the introduction or emergence of new strains with more invasive or epidemic potential in the population.

## Figures and Tables

**Table 1:** Isolation sites of confirmed and probable iGAS cases (n = 22).

Site	N	Percentage (%)
**Blood**	7	31.8
**Pleural fluid**	7	31.8
**Pus from deep abscess**	4	18.2
**Pus from mastoid surgery**	2	9.1
Pus from perforated ear *	1	4.5
Throat swab *	1	4.5
Synovial fluid **	1	4.5

* Two of the above were probable cases. ** In one case blood as well as synovial fluid culture were positive.

**Table 2:** Clinical syndromes of iGAS cases and outcome.

Patient	Age (mo)	Clinical Syndromes	Outcome
**1**	20	Scarlet fever, complicated pneumonia	ICU-Recovered
**2**	41	Bacteraemia with endocarditis	Recovered
**3**	104	Bacteraemia, otitis, perforation of ear drum, mastoiditis, meningitis	Recovered
**4**	47	Bacteraemia, cellulitis, fasciitis	Recovered
**5**	52	Complicated pneumonia, STSS	ICU-ECMO-Recovered
**6**	26	Bacteraemia, STSS	ICU-Died
**7**	17	Bacteraemia, STSS	ICU-Died
**8**	46	Bacteraemia, joint infection	Recovered
**9**	119	Deep abscess and fasciitis extending to pelvic floor muscles	Recovered
**10**	24	Bacteraemia, scarlet fever, sepsis, complicated pneumonia, STSS	ICU-Recovered
**11**	55	Bacteraemia, scarlet fever, otitis media, perforation of ear drum	Recovered
**12**	12	Otitis media, perforation of ear drum, mastoiditis	Recovered
**13**	65	Otitis media, mastoiditis, sigmoid sinus thrombosis	Recovered
**14**	85	Scarlet fever, otitis media, complicated pneumonia	ICU-Recovered
**15**	24	Scarlet fever, complicated pneumonia	Recovered
**16**	90	Fever, cough, complicated pneumonia	Recovered
**17**	78	Fever, cough, complicated pneumonia	Recovered
**18**	80	Scarlet fever, complicated pneumonia, STSS	ICU-Recovered
**19**	15	Fever, diarrhoea, complicated mastoiditis	Recovered
**20**	101	Fever, pharyngitis, intraorbital abscess	Recovered
**21**	14	Otitis media, complicated mastoiditis	Recovered
**22**	76	Fever, drowsiness, retropharyngeal abscess	Recovered

**Table 3:** *emm* type distribution of iGAS strains.

*Emm* Type	Number	Percentage (%)
*emm* 1.0	6	66.67
*emm* 92.0	1	11.11
*emm* 2.0	1	11.11
*emm* 1.150	1	11.11

## Data Availability

The original contributions presented in this study are included in the article. Further inquiries can be directed to the corresponding author.

## References

[1] Stevens D.L. (1992). Invasive Group A Streptococcus Infections. Clin. Infect. Dis..

[2] Miller K.M., Lamagni T., Cherian T., Cannon J.W., Parks T., Adegbola R.A., Pickering J., Barnett T., Engel M.E., Manning L. (2022). Standardization of Epidemiological Surveillance of Invasive Group A Streptococcal Infections. Open Forum Infect. Dis..

[3] Stevens D.L., Tanner M.H., Winship J., Swarts R., Ries K.M., Schlievert P.M., Kaplan E. (1989). Severe Group A Streptococcal Infections Associated with a Toxic Shock-like Syndrome and Scarlet Fever Toxin A. N. Engl. J. Med..

[4] Efstratiou A., Lamagni T. (2022). Epidemiology of Streptococcus Pyogenes. Streptococcus Pyogenes: Basic Biology to Clinical Manifestations.

[5] de Gier B., Marchal N., de Beer-Schuurman I., te Wierik M., Hooiveld M., de Melker H.E., van Sorge N.M. (2023). Increase in Invasive Group A Streptococcal (Streptococcus Pyogenes) Infections (IGAS) in Young Children in the Netherlands, 2022. Eurosurveillance.

[6] Johannesen T.B., Munkstrup C., Edslev S.M., Baig S., Nielsen S., Funk T., Kristensen D.K., Jacobsen L.H., Ravn S.F., Bindslev N. (2023). Increase in Invasive Group A Streptococcal Infections and Emergence of Novel, Rapidly Expanding Sub-Lineage of the Virulent Streptococcus Pyogenes M1 Clone, Denmark, 2023. Eurosurveillance.

[7] Barnes M., Youngkin E., Zipprich J., Bilski K., Gregory C.J., Dominguez S.R., Mumm E., McMahon M., Como-Sabetti K., Lynfield R. (2023). Notes from the Field: Increase in Pediatric Invasive Group A Streptococcus Infections—Colorado and Minnesota, October–December 2022. MMWR Morb. Mortal. Wkly. Rep..

[8] van Kempen E.B., Bruijning-Verhagen P.C.J., Borensztajn D., Vermont C.L., Quaak M.S.W., Janson J.-A., Maat I., Stol K., Vlaminckx B.J.M., Wieringa J.W. (2023). Increase in Invasive Group a Streptococcal Infections in Children in the Netherlands, A Survey Among 7 Hospitals in 2022. Pediatr. Infect. Dis. J..

[9] Aboulhosn A., Sanson M.A., Vega L.A., Segura M.G., Summer L.M., Joseph M., McNeil J.C., Flores A.R. (2023). Increases in Group A Streptococcal Infections in the Pediatric Population in Houston, TX, 2022. Clin. Infect. Dis..

[10] Kizil M.C., Kara Y., Bozan G., Arda S., Durmaz G., Kilic O., Dinleyici E.C. (2023). Consecutive Seven Serious Cases with Invasive Group A Streptococcal Infections at December 2022–January 2023. Pediatr. Infect. Dis. J..

[11] Statistical Services Population—Publications. https://www.cystat.gov.cy/en/PublicationList?s=46&utm_source=chatgpt.com.

[12] Statistical Service & Press and Information Office Census of Population and Housing 2021: Final Results. https://www.gov.cy/en/economy-and-finance/census-of-population-and-housing-2021-final-results/?utm_source=chatgpt.com.

[13] Breiman R.F., Davis J.P., Facklam R.R. (1993). Defining the Group A Streptococcal Toxic Shock Syndrome. Rationale and Consensus Definition. JAMA.

[14] EUCAST Breakpoints 2022: Clinical Breakpoints and Dosing of Antibiotics. https://www.eucast.org/clinical_breakpoints.

[15] Darenberg J., Luca-Harari B., Jasir A., Sandgren A., Pettersson H., Schalen C., Norgren M., Romanus V., Norrby-Teglund A., Normark B.H. (2007). Molecular and Clinical Characteristics of Invasive Group A Streptococcal Infection in Sweden. Clin. Infect. Dis..

[16] Vieira A., Wan Y., Ryan Y., Li H.K., Guy R.L., Papangeli M., Huse K.K., Reeves L.C., Soo V.W.C., Daniel R. (2024). Rapid Expansion and International Spread of M1UK in the Post-Pandemic UK Upsurge of Streptococcus Pyogenes. Nat. Commun..

[17] The R Project for Statistical Computing. https://www.r-project.org/.

[18] Wickham H. (2009). Ggplot2.

[19] Zakikhany K., Degail M.A., Lamagni T., Waight P., Guy R., Zhao H., Efstratiou A., Pebody R., George R., Ramsay M. (2011). Increase in Invasive Streptococcus Pyogenes and Streptococcus Pneumoniae Infections in England, December 2010 to January 2011. Eurosurveillance.

[20] Guy R., Henderson K.L., Coelho J., Hughes H., Mason E.L., Gerver S.M., Demirjian A., Watson C., Sharp A., Brown C.S. (2023). Increase in Invasive Group A Streptococcal Infection Notifications, England, 2022. Eurosurveillance.

[21] Davies M.R., Keller N., Brouwer S., Jespersen M.G., Cork A.J., Hayes A.J., Pitt M.E., De Oliveira D.M.P., Harbison-Price N., Bertolla O.M. (2023). Detection of Streptococcus Pyogenes M1UK in Australia and Characterization of the Mutation Driving Enhanced Expression of Superantigen SpeA. Nat. Commun..

[22] Demczuk W., Martin I., Domingo F.R., MacDonald D., Mulvey M.R. (2019). Identification of Streptococcus Pyogenes M1UK Clone in Canada. Lancet Infect. Dis..

[23] Ekelund K., Skinhøj P., Madsen J., Konradsen H.B. (2005). Reemergence of Emm 1 and a Changed Superantigen Profile for Group A Streptococci Causing Invasive Infections: Results from a Nationwide Study. J. Clin. Microbiol..

[24] Dabaja-Younis H., Kandel C., Green K., Johnstone J., Zhong Z., Kassee C., Allen V., Armstrong I., Baqi M., Barker K. (2025). Invasive Group A Streptococcal Infection in Children, 1992–2023. JAMA Netw. Open.

[25] Group A Streptococcal Infections: Report on Seasonal Activity in England, 2022 to 2023. https://www.gov.uk/government/publications/group-a-streptococcal-infections-activity-during-the-2022-to-2023-season/group-a-streptococcal-infections-report-on-seasonal-activity-in-england-2022-to-2023#references.

[26] Koliou M., Ioannou Y., Efstratiou A., Hannidou N., Pieri V., Alexandrou M., Soteriades E.S. (2007). Circulating Serotypes and Antimicrobial Sensitivity of Streptococcus Pyogenes Isolates from Children in Cyprus. Clin. Microbiol. Infect..

[27] ECDC (2018). Invasive Pneumococcal Disease Annual Epidemiological Report for 2018 Key Facts.

[28] Abo Y.-N., Oliver J., McMinn A., Osowicki J., Baker C., Clark J.E., Blyth C.C., Francis J.R., Carr J., Smeesters P.R. (2023). Increase in Invasive Group A Streptococcal Disease among Australian Children Coinciding with Northern Hemisphere Surges. Lancet Reg. Health West. Pac..

[29] Rümke L.W., de Gier B., Vestjens S.M.T., van der Ende A., van Sorge N.M., Vlaminckx B.J.M., Witteveen S., van Santen M., Schouls L.M., Kuijper E.J. (2020). Dominance of M1UK Clade among Dutch M1 Streptococcus Pyogenes. Lancet Infect. Dis..

[30] Willyard C. (2022). Flu and Colds Are Back with a Vengeance—Why Now?. Nature.

[31] McCullers J.A. (2006). Insights into the Interaction between Influenza Virus and Pneumococcus. Clin. Microbiol. Rev..

[32] Morens D.M., Taubenberger J.K., Fauci A.S. (2008). Predominant Role of Bacterial Pneumonia as a Cause of Death in Pandemic Influenza: Implications for Pandemic Influenza Preparedness. J. Infect. Dis..

